# Sinusitis: A Rare Cause for Galactorrhoea

**DOI:** 10.1155/2012/981375

**Published:** 2012-11-13

**Authors:** W. O. Bennett, J. R. Kennedy, V. M. Reddy, R. Dyer, S. A. Hickey

**Affiliations:** ^1^Department of Otolaryngology-Head and Neck Surgery, Torbay Hospital, South Devon Healthcare NHS Foundation Trust, Torquay TQ2 7AA, UK; ^2^Department of Endocrinology, Torbay Hospital, South Devon Healthcare NHS Foundation Trust, Torquay TQ2 7AA, UK

## Abstract

A 32-year-old woman presented to the endocrinology clinic with recent onset galactorrhoea. Investigations revealed raised prolactin levels. An MRI scan demonstrated a normal pituitary gland, and an incidental finding of sphenoid sinusitis with expansion of the sphenoid sinus was thought to be due to a mucocele. It is postulated that either the direct local pressure by the mucocele or localised inflammation secondary to sinusitis might cause hyperprolactinaemia. The patient underwent endoscopic surgery to drain the mucocele, after which her galactorrhoea resolved. A review of the literature reveals only one previously documented case of sinusitis causing hyperprolactinaemia and galactorrhoea.

## 1. Introduction

Sinusitis is a common condition: the average primary care physician may see around 50 cases per year [1]. The majority are viral in origin with only 2% complicated by bacterial sinusitis [1, 2]. Sinusitis may be complicated by orbital, endocranial, and osseous extensions [3].

Galactorrhoea is common, affecting 20 to 25 percent of women at some time in their life [2] and may have a variety of causes from the normal physiological state to the sequelae of malignant disease [4, 5]. It is a manifestation of hyperprolactinaemia, which is usually due to a secretory pituitary adenoma, or loss of normal dopamine-mediated inhibition of prolactin release due to compression of the pituitary or its stalk.

We describe a rare case of galactorrhoea and hyperprolactinaemia secondary to sphenoid sinus disease. There has only been one similar case reported in the English language medical literature, which described an erosive sphenoid sinusitis presenting as bilateral optic neuritis in association with galactorrhoea and hyperprolactinaemia [6]. 

## 2. Case Report

A 32-year-old lady presented to the endocrinology clinic with a recent onset of galactorrhoea. Her serum prolactin level was 1168 mu/L (normal range 102–496 mu/L). Other pituitary hormone levels and thyroid function were normal. A pregnancy test was negative. The patient reported no ophthalmic symptoms and was taking no drugs likely to cause hyperprolactinaemia.

**Figure 1:**
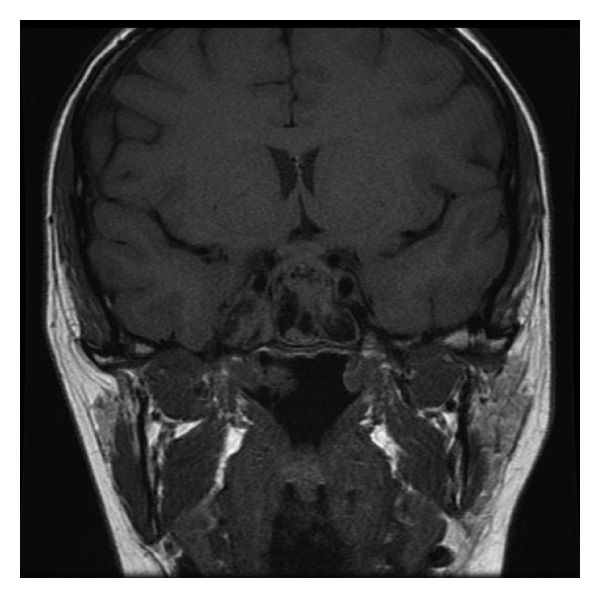
Coronal MRI image showing expanded sphenoid sinus with a suspected mucocele.

**Figure 2:**
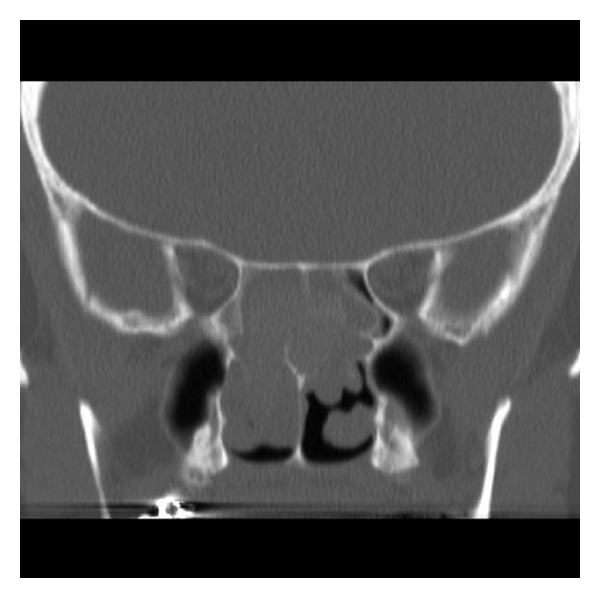
Coronal CT image showing bilateral opacification and polyposis in both sphenoethmoidal recesses.

A magnetic resonance imaging (MRI) scan of the head demonstrated no abnormality of the pituitary gland, but revealed an expanded sphenoid sinus with a mucocele (Figure 1). There was loss of the normal sella turcica anatomy and extension into the ethmoid air cells. The findings prompted referral to an ENT surgeon. A CT scan of the sinuses demonstrated bilateral concha bullosa and polyposis in the sphenoethmoidal recess obstructing the sphenoid sinus ostium (Figure 2). 

The patient underwent endoscopic sinus surgery involving bilateral nasal polypectomy and sphenoidotomies. The sphenoid sinus contained polypoid mucosa and thick, elastic mucus, raising suspicions of fungal infection (neither bacteria nor fungi were grown on culture subsequently). She was discharged with a two-week course of empirical oral co-amoxiclav.

In the immediate postoperative period, she continued to complain of galactorrhoea and nasal obstruction. A noncontrast CT scan showedhighattenuationmaterial in the sphenoid sinus,suggesting residualfungalinfection. Three months after the operation her galactorrhoea had resolved, and she had no nasal symptoms. A repeat CT scan showed bilateral opacification of the ethmoid and sphenoid sinuses. Examination revealed recurrent nasal polyposis and green mucopus in the right sphenoethmoidal recess. Revision right nasal polypectomy was carried out, and similar debris was removed from the right sphenoid sinus.

The patient was discharged on intranasal steroids and a course of clarithromycin. Swabs from the right sphenoid sinus grew Haemophilus influenze, with no evidence of fungal infection. 

At thirteen months followup the patient remained asymptomatic with no further episodes of galactorrhoea. Her prolactin level returned to within normal limits (228 mu/L). A follow up CT scan showed no residual disease. Endoscopy revealed healthy aerated sphenoid sinuses. 

## 3. Discussion

In this case, the surgical management of sphenoiditis with sphenoid mucocele resulted in the resolution of otherwise unexplained galactorrhoea. We suggest that the pituitary dysfunction was sinogenic due to local pressure effect or inflammation. As with the previous case report (Khokhar et al., 2002), the local pressure effect or inflammation may have disrupted the inhibitory dopaminergic axis between the hypothalamus and the pituitary gland causing hyperprolactinaemia and galactorrhoea [6]. The resolution of symptoms following surgery strengthens this hypothesis. 

Prolactin is a 199 amino acid protein produced in the lactotroph cells of the anterior pituitary gland [7]. Its primary function is to enhance breast development during pregnancy and induce lactation [8]. Prolactin secretion is controlled primarily by inhibition from the hypothalamus, mainly by dopamine but also to a lesser extent by gamma-aminobutyric acid (GABA) and somatostatin [9].

Hyperprolactinaemia is usually thought to be caused by either disinhibition (due to compression of the pituitary stalk and reduced dopamine levels) or excess production from a prolactinoma (a pituitary gland adenoma). In some cases, the cause is not clear. It is estimated to affect approximately 1% of females [9]. Gynaecologists manage most cases as patients commonly present with amenorrhoea and infertility. Adenomas less than 5 mm in diameter may not be seen on an MRI or CT, scan, and in such cases patient may be labelled as having idiopathic hyperprolactinaemia [9]. MRI is more sensitive at detecting pituitary lesions than CT in contrast. 

Sinusitis has been described as causing optic neuritis [6, 10] or orbital apex syndrome (OAS) [11], and in one isolated case was thought to be the cause of galactorrhoea and hyperprolactinaemia [6]. Ethmoid sinus disease may spread beyond the thin lamina papyracea bony wall to the orbit, which can cause unilateral or bilateral optic neuritis by virtue of the close proximity to the optic nerves [6, 11]. OAS presents with visual loss and ophthalmoplegia, but with minimal or absent signs of orbital inflammation [11]. It has also been termed posterior orbital cellulitis [10]. Both our case and the previous case had erosion of the floor of the sella turcica, which we presume leads to irritation or compression of the pituitary stalk. This case differs from the previous literature in terms of the absence of clinical optic neuritis and also the presence of a mucocele in the sphenoid sinus. Surgical drainage of sinus disease has been shown to result in rapid and permanent relief of symptoms in all of both cases [6].
